# NanoUPLC-QTOF-MS/MS Determination of Major Rosuvastatin Degradation Products Generated by Gamma Radiation in Aqueous Solution

**DOI:** 10.3390/ph14111160

**Published:** 2021-11-13

**Authors:** Lucija Dončević, Ema Svetličić, Amela Hozić, Branka Mihaljević, Dorota Jarmużek, Ivana Tartaro Bujak, Donata Pluskota-Karwatka, Luka Ozdanovac, Iva Džeba, Mario Cindrić

**Affiliations:** 1 Division of Molecular Medicine, Ruđer Bošković Institute, Bijenička 54, 10000 Zagreb, Croatia; ldoncev@irb.hr (L.D.); ahozic@irb.hr (A.H.); 2 Department of Biochemical Bioengineering, Faculty of Food Technology and Biotechnology, University of Zagreb, Pierottijeva 6, 10000 Zagreb, Croatia; esvetlicic@pbf.hr; 3 Division of Materials Chemistry, Ruđer Bošković Institute, Bijenička 54, 10000 Zagreb, Croatia; mihozeg@irb.hr (B.M.); itartaro@irb.hr (I.T.B.); idzeba@irb.hr (I.D.); 4 Faculty of Chemistry, Adam Mickiewicz University in Poznan, Uniwersytetu Poznanskiego 8, 61-614 Poznan, Poland; dorota.jarmuzek@amu.edu.pl (D.J.); donatap@amu.edu.pl (D.P.-K.); 5 Research and Development Ltd., PLIVA, Prilaz Baruna Filipovića 29, 10000 Zagreb, Croatia; luka.ozdanovac@pliva.com

**Keywords:** rosuvastatin, nanoLC-MS, gamma radiation, degradation products identification, OH radical mechanisms

## Abstract

Rosuvastatin, a member of the statin family of drugs, is used to regulate high cholesterol levels in the human body. Moreover, rosuvastatin and other statins demonstrate a protective role against free radical-induced oxidative stress. Our research aimed to investigate the end-products of free radical-induced degradation of rosuvastatin. To induce the radical degradation, an aqueous solution of rosuvastatin was irradiated using different doses of gamma radiation (50–1000 Gy) under oxidative conditions. Rosuvastatin and related degradation products were separated on nanoC18 column under gradient elution, and identification was carried out on hyphenated nanoUPLC and nanoESI-QTOF mass spectrometer system. Elemental composition analysis using highly accurate mass measurements together with isotope fitting algorithm identified nine major degradation products. This is the first study of gamma radiation-induced degradation of rosuvastatin, where chemical structures, MS/MS fragmentation pathways and formation mechanisms of the resulting degradation products are detailly described. The presented results contribute to the understanding of the degradation pathway of rosuvastatin and possibly other statins under gamma radiation conditions.

## 1. Introduction

Rosuvastatin (RSV), chemically described as (*E*)-7-(4-(4-fluorophenyl)-6-isopropyl-2 -(*N*-methylmethylsulfonamide)pyrimidin-5-yl)-3,5-dihydroxyhept-6-enoic acid, is a member of the class of statins [1]. Statins are effective cholesterol-lowering drugs, most commonly prescribed in the treatment of hyperlipidemia, mixed dyslipidemia, and related conditions [2]. In general, statins act as inhibitors of 3-hydroxy-3-methylglutaryl coenzyme A (HMG-CoA) reductase, which is a rate-limiting enzyme in cholesterol synthesis, converting HMG-CoA to mevalonate [3]. Moreover, previous studies have demonstrated the antioxidant properties of RSV. It was hypothesized that RSV and other statins have a protective role against oxidative stress by reducing NAD(P)H oxidase and upregulating antioxidant enzyme superoxide dismutase [4,5]. In general, oxidative stress is defined as an imbalance between free radical reactions and a lack of defense against these reactions in living organisms. Among all free radicals, ^●^OH radical is one of the most reactive oxygen species (ROS) [6]. In addition to research of statin antioxidant potential in vivo [7,8,9], previous studies have also shown that statins could act against hydroxyl radicals generated in aqueous solutions [10]. Since large accumulation of reactive oxygen species and free radicals increases the risk for various cardiovascular and cerebrovascular diseases, the use of statins is also encouraged to prevent the occurrence of named diseases. One of the successful methods for the generation of free radicals in aqueous solutions is gamma radiolysis. The result of gamma irradiation of aqueous solution is water radiolysis described as [11,12]:

H_2_O ⇝ (0.28) ^●^OH + (0.06) H^●^ + (0.27) e^−^*_aq_* + (0.05) H_2_ + (0.07) H_2_O_2_ + (0.27) H^+^ (1)

where the number in parentheses represents the radiolytic yield or G-value in µmol/J. Saturation of the solution with nitrous oxide quantitatively converts the aqueous electrons and hydrogen radicals to the ^●^OH radicals according to reactions presented in Equations (2) and (3) [12,13]. Thus, gamma radiolysis under oxidative conditions enables the study of only the ^●^OH radical reactions.

e^−^_aq_ + N_2_O + H_2_O → N_2_ + ^−^OH + ^●^OH (2)

H^●^ + N_2_O → ^●^OH + N_2_ (3)

The effect of gamma radiation-induced oxidation has previously been studied on various drugs to understand their antioxidant mode of action [14,15]. Furthermore, the degradation of pharmaceuticals caused by gamma radiation can also be used for remediation of drinking water and wastewater [16,17,18]. Since statins are widely consumed pharmaceuticals, they accumulate in wastewater and sewage worldwide and thus represent potential bioactive hazard for human health or animal and plant ecosystem [19,20,21,22]. It was reported earlier that rosuvastatin does not extensively metabolize in the human body and 77% of it excretes as unchanged. Correspondingly, rosuvastatin can reach concentrations of 552 ng/L in effluent and 604 ng/L in influent samples of sewage [19]. Consequently, from the ecological point of view, it is important to identify and characterize degradation products formed due to interactions with reactive radicals induced by gamma radiation of RSV aqueous solution.

A previous study has proposed reaction kinetics and degradation mechanisms of several statins following Cs-137 gamma irradiation [23]. To the best of our knowledge, no such studies have been conducted on RSV. However, a large number of analytical techniques were used for the identification and structural characterization of RSV under different stress conditions such as extreme pH and temperature, humidity, photoirradiation, and others [24,25,26]. Related impurities and metabolites of RSV were analyzed by RP-UPLC [24,27], LC-MS/MS [28], LC-MS/TOF [29] and with a combination of hyphenated analytical techniques like LC-MS^n^, LC-NMR, and H/D exchange [30] in order to deduce structural changes and degradation mechanisms of named molecules in aqueous solutions. A valuable insight into the structural characterization was gained through the use of micro- and nano-analytical chromatographic techniques in a sense of increased sensitivity and separation efficacy, and reduced sample consumption [31]. Although LC-MS is widely accepted method for the structural elucidation of unknown structures, even accurate mass measurement sometimes cannot discriminate molecules with a similar mass and composition [32]. Thus, for the unambiguous structural determination of unknown analytes, LC-MS can be coupled to methods such as Nuclear Magnetic Resonance (NMR) [33].

The main objectives of this work were to investigate the degradation of an aqueous solution of RSV affected by gamma radiation at different radiation doses, identify produced degradation molecules, and suggest mechanisms of their formation. Several degradative molecular mechanisms were interconnected with already familiar RSV or other statin degradation products formed in forced stability testing, metabolic degradation, or oxidative stress pathways [24,25,26,27,28,30,34]. While four of the nine detected degradation products were already known from the literature, in this study five previously unknown structures of RSV degradation products are described for the first time in detail with corresponding fragmentation pathways and formation mechanisms. Chromatographic and mass spectrometry nano-techniques were employed in this research to explicate the interaction of RSV with hydroxyl radicals under forced degradation conditions. Although nano-LC techniques were employed in the previous research for the purpose of RSV degradation product elucidation [34], the herein described use of high resolution (>20,000) mass spectrometer enabled the identification of large number of molecules in solely one MS and MS/MS analysis. Moreover, the highly accurate mass measurement with mass error of less than 2 ppm together with isotope-fitting algorithm provided the accurate prediction and calculation of unknown molecular structures without pairing to other analytical techniques.

## 2. Results and Discussion

### 2.1. Chromatographic Analysis of Irradiated Samples

**Figure 1:**
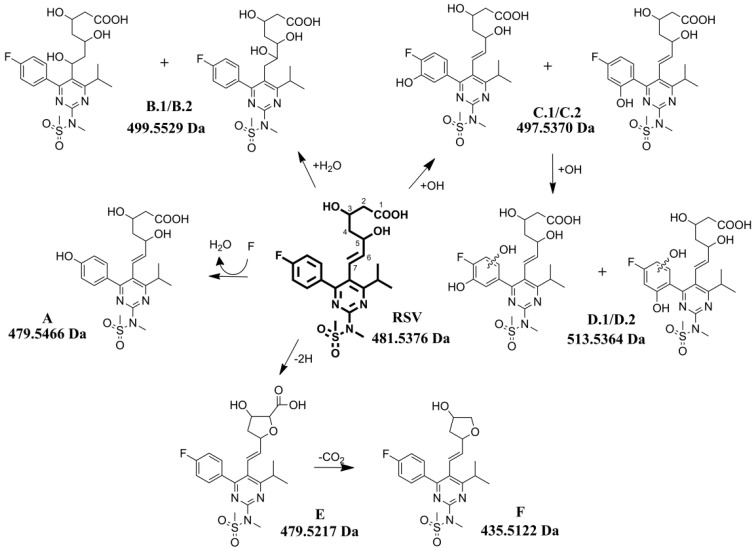
Structures and belonging molecular weights in Da of rosuvastatin (RSV) molecule and degradation products induced by radiation dose up to 1000 Gy: A, B.1/B.2, C.1/C.2, D.1/D.2, E, and F.

**Figure 2:**
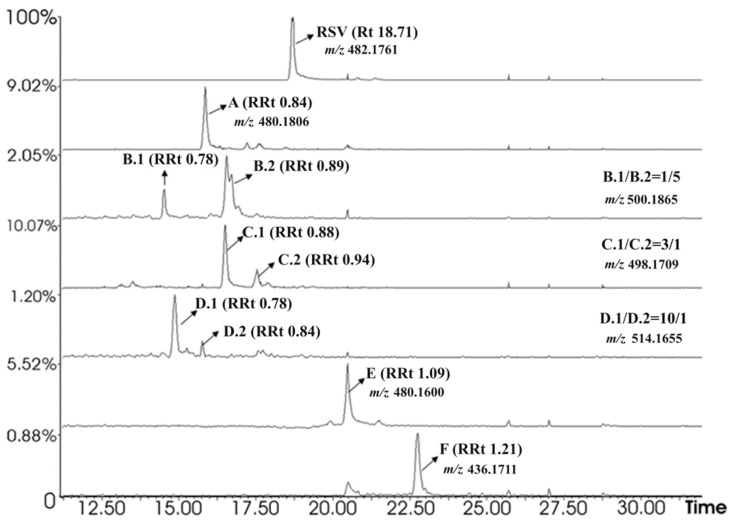
Extracted ion chromatograms of RSV (Rt 18.71) and gamma radiation-induced degradation products at 1000 Gy with observed relative retention time (RRt). Intensities of degradation products are normalized to the RSV principal peak. *Peak areas* of degradation peaks are expressed as *percent* area of the RSV principal peak. Fraction of extracted peak masses of B.1/B.2, C.1/C.2, and D.1/D.2 is calculated by dividing isobaric peak areas.

Irradiated and non-irradiated RSV samples were analyzed under the same chromatographic and mass spectrometry conditions to estimate differences between non-irradiated RSV and RSV samples irradiated at doses of 50, 100, 200, 500, and 1000 Gy (Figure S1 in Supplementary Materials). A total of nine degradation products emerged after gamma irradiation of RSV molecule. Molecular structures of RSV forced degradation products are shown in Figure 1. Extracted ion chromatograms of irradiated samples at gamma radiation doses of 50 and 100 Gy implied production of seven degradation products A, C.1/C.2, D.1/D.2, E, and F. Further analysis of samples irradiated at 200, 500, and 1000 Gy showed the production of two additional degradation products B.1/B.2 (Figure 2). The radiation doses were selected according to the literature [16,17,18] concerning gamma remediation of wastewater where the dose of 1000 Gy was the most applied radiation dose for the remediation of pharmaceutical contaminated water. Although the obtained results of our research provide qualitative information about the structure of rosuvastatin degradation products, the design of the remediation process should aim to completely decompose the pollutant without creating harmful intermediates. Furthermore, complete removal of pollutants and/or harmful substances due to the diverse composition of water and toxicity is almost impossible to achieve using only one method. Therefore, a combination of methods such as ionizing radiation with for example H_2_O_2_, ozonation or TiO_2_ nanoparticles could improve the degradation efficacy [35,36].

### 2.2. Fragmentation Pathway of RSV

RSV with molecular formula C_22_H_29_N_3_O_6_FS and theoretical monoisotopic mass *m/z* 482.1761 was eluted at 18.71 min in the total ion chromatogram (TIC), as shown in Figure 2. The fragmentation of RSV was previously conducted using an Imaging MALDI-TOF mass spectrometer where three major product ions were formed (*m/z* 300, 272, and 258) and it was proposed that the protonation occurs in the nitrogen of the *N*-methylmethanesulfonamide of RSV molecule [37]. Detailed fragmentation study was performed using LC-QTOF-MS in ESI positive ion mode where the characteristic fragmentation ions of *m/z* 464, 446, 422, 404, 344, 300, 272, and 270 were observed [29,30]. The authors proposed similar fragmentation pathway as shown in our results and described below.

**Figure 3:**
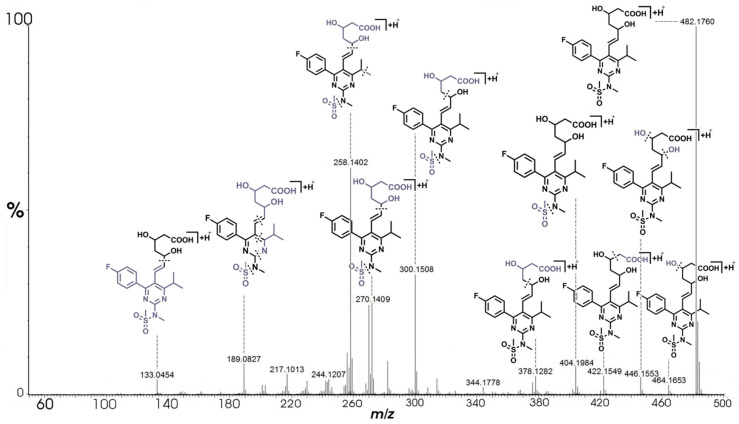
Fragmentation MS/MS spectra of RSV [M + H]^+^ ion (precursor ion mass *m/z* 482.1760) obtained by using collision energy of 25 V.

The fragmentation pathway of RSV (Figure 3) was induced by the elimination of the water molecule related to the C-3 and C-5 atoms (*m/z* 482.1760 → *m/z* 464.1653 → *m/z* 446.1553). The fragment ion at *m/z* 422.1549 was generated by the loss of −C_2_H_4_O_2_, whereas the fragment ion at *m/z* 404.1984 was formed by the loss of methyl sulfonyl group. Fragment ion at *m/z* 378.1282 was formed by the cleavage between C-5 and C-4 atoms. The same ion with additional loss of methyl sulfonyl group formed the fragment at *m/z* 300.1508. Ions at *m/z* 270.1409, 258.1402, and 217.1013 were produced by the loss of methyl sulfonyl group and −CH(OH)CH_2_CH(OH)CH_2_COOH at C-5 position with the extra cleavage of methyl group and isopropyl group of fragments at *m/z* 258.1402 and 217.1013, respectively. The ion at *m/z* 189.0827 was produced by the loss of methyl sulfonyl group, the cleavage between C-6 and C-7, and additional internal cleavages of the pyrimidine ring. The last significant fragment ion in the mass spectrum at *m/z* 133.0454 corresponds to the non-conjugated part of RSV molecule with molecular formula C_5_H_9_O_4_. A fragmentation pattern of RSV served as a starting point to deduce sub-structural information on degradation products A, B.1/B.2, C.1/C.2, D.1/D.2, E, and F after the comparison of RSV and RSV degradation products MS/MS spectra.

### 2.3. Characterization of Degradation Products

Analysis of TIC of irradiated and non-irradiated RSV samples revealed differences in the chromatograms and consequently in subtracted MS spectra. The nine precursor ions observed in the spectra of irradiated RSV samples were considered to originate from degradation products and used for subsequent accurate MS/MS analysis.

Previously, various methods were used to induce forced degradation of RSV where its impurities and degradation products were tentatively identified and analyzed. However, only a few studies looked into more detailed structural characteristics of the resulting products [29,30,34,38,39]. Rosuvastatin was found highly sensitive to UV degradation where two major products emerged with the same *m/z* as the drug. The products were characterized as polycyclic isomers of the drug because the formation of polycyclic benzoquinazoline isomers was previously reported. In the same study a degradation product having *m/z* of 498 was also formed by photodegradation, the authors suggested it to be rosuvastatin-N-oxide [1]. Although the product of *m/z* 498 was found in our research (products C.1/C.2), the MS/MS pattern did not match the named molecule. In the study by Shah et al. [30], a total of 11 RSV degradation products were formed under hydrolytic and photolytic conditions. The LC-MS analysis showed that five degradation products had the same *m/z* as RSV and six degradation products had the mass which was 18 Da lower than the drug. The proposed mechanism for acid hydrolysis is esterification of RSV to lactone which then undergoes hydrolysis and converts back to the drug together with its diastereoisomer. The main pathway of RSV under photolytic conditions was found to be cyclization reaction through 1,5-hydrogen shift [30]. Degradation products A, C.1/C.2, and E were earlier reported to be produced during photocatalysis of RSV with ZnO [29], although the structure of product E was described only at the level of molecular formula, and the C.1/C.2 isomers were not distinguished because the position of the hydroxyl substituent in the fluorophenyl ring was not determined. Degradation products B.1/B.2, D.1/D.2, and F along with the proposed formation mechanisms were not previously described in the literature. Experimental mass, calculated molecular formula, theoretical mass, RDB, and MS/MS fragment ions of RSV and degradation products are shown in Table 1.

#### 2.3.1. Degradation Product A (*m/z* 480.1805)

**Figure 4:**
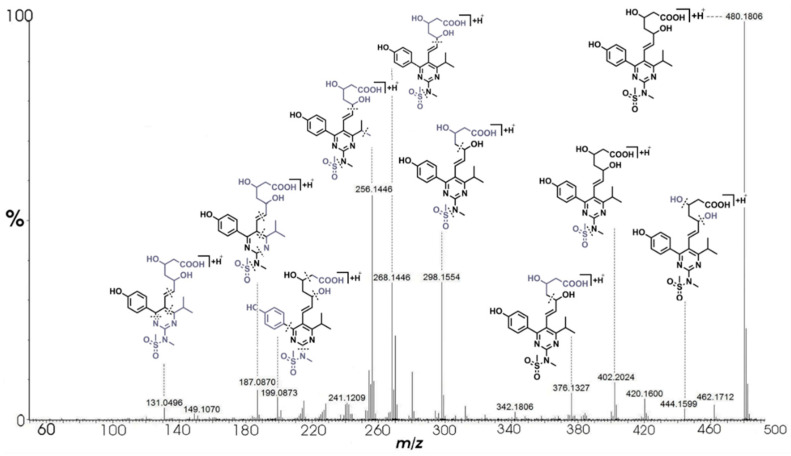
Fragmentation MS/MS spectra of product A [M + H]^+^ ion (precursor ion mass *m/z* 480.1806) obtained by using collision energy of 25 V.

**Scheme 1:**
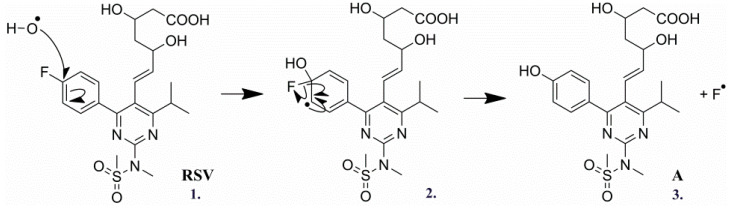
Proposed mechanism of the compound A formation by radical substitution on RSV.

Compound A was eluted and detected at relative retention time (RRt) 0.84 in the TIC (Figure 2). The compound [M + H]^+^ ion was observed at *m/z* 480.1806, which corresponds to the elemental composition of C_22_H_30_N_3_O_7_S. The proposed molecular formula of compound A differs from the RSV formula (C_22_H_29_N_3_O_6_FS) by the presence of additional atoms: oxygen and hydrogen, and the absence of a fluorine atom. As it is presented in Figure 4, the MS/MS analysis of degradation product A showed the same fragmentation pattern as RSV with the mass shift of 1.9957 Da on average, except for two fragments detected in the low mass region (*m/z* 199.0873 and 131.0496). Mass differences and fragmentation pattern indicated that the molecule of product A contains a hydroxyl group at the position of the fluorine substituent. The average mass shift of 1.9956 Da complements the mass difference of fluorine and hydroxyl radical, 18.9984 Da and 17.0027 Da, respectively. Therefore, it is proposed that compound A resulted by radical substitution of fluorine atom on RSV molecule (Scheme 1) and the chemical name is proposed as (*E*)-7-(4-(4-hydroxyphenyl)-6-isopropyl-2-(*N*-ethylmethylsulfonamide)pyrimidin-5-yl)-3,5-dihydroxyhept-6-enoic acid.

#### 2.3.2. Degradation Products B.1/B.2 (*m/z* 500.1867)

**Scheme 2:**
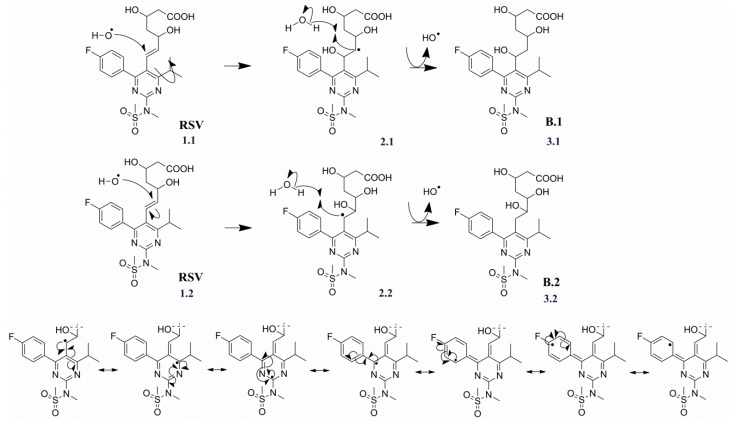
Proposed mechanism of the compounds B.1 and B.2 formation by radical hydroxylation of RSV. Resonance structures showing stabilization of the intermediate product leading to the compound B.2 generation.

Compounds B.1 and B.2 were eluted and detected at RRt 0.78 and RRt 0.89, respectively, as it is shown on the TIC (Figure 2). Protonated ions [M + H]^+^ of both products were observed at *m/z* 500.1865, corresponding to the elemental composition of C_22_H_31_N_3_O_7_FS. Such identical molecular formulas indicate that B.1 and B.2 are a pair of isomers that differ from RSV in the presence of a water molecule. Therefore, it was proposed that the compounds resulted from the radical hydroxylation of the double bond between C-6 and C-7 (Scheme 2). In the previous study, it was reported that the addition of hydroxyl radical was favored to the position C-6 [29]. Our results are consistent with this finding. Compound B.2 resulted from the addition of hydroxyl radical to C-6 and is the major regioisomer formed (Figure 2). We expected such reactivity which is obvious because the addition to C-6 leads to a more stable intermediate formation (Scheme 2 (2.2)). The stabilization is provided by the resonance with the π electrons of the fluorophenyl and the pyrimidine rings (Scheme 2). When hydroxyl radical undergoes addition to C-7, only resonance with the π electrons of the fluorophenyl ring contributes to the intermediate product stabilization. However, hydroxyl radicals generated by water radiolysis often react non-selectively. Therefore, radical hydroxylation of the C-6=C-7 bond was not regioselective and B.1, which resulted from the addition to C-7, was also formed as a minor regioisomer (Figure 2). Extracted ion chromatogram of product B.2 displayed the double chromatographic peak at RRt 0.89 (Figure 2). Such observation indicated the co-elution of two compounds of the same molecular mass. Moreover, radical hydroxylation of RSV leads to the formation of an additional chiral center. It was assumed that the double peak emerged due to a mixture of two diastereoisomers of product B.2. Consequently, both peak areas at RRt 0.89 were taken into account for the measurement of B.1/B.2 ratio.

Furthermore, degradation products B.1 and B.2 were not formed when the smallest dosage of radiations were applied (50 and 100 Gy). This observation can be explained by the mechanistic comparison with degradation products: A, C.1, C.2, D.1, and D.2 that were formed by the substitution of hydroxyl radical with a hydrogen atom bonded to the highly conjugated benzene ring. In comparison to that, compounds B.1/B.2 were produced by the addition of hydroxyl radical to the C-6=C-7 bond, which concludes that the radical substitution reaction occurs more readily than the radical addition reaction.

**Figure 5:**
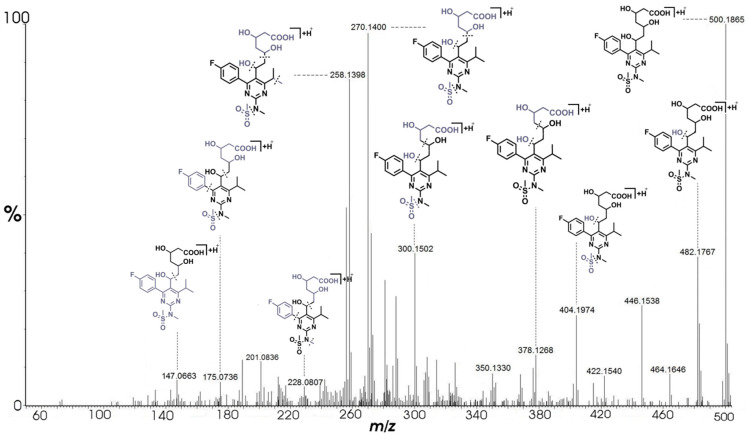
Fragmentation MS/MS spectra of product B.1 [M + H]^+^ ion (precursor ion mass *m/z* 500.1865) obtained by using collision energy of 25 V.

The formation of B.1 in difference to B.2 was confirmed by diagnostic fragments *m/z* 175.0736 and 147.0663 as seen in the MS/MS spectrum of the hydroxylation product B.1 (Figure 5). Besides two named diagnostic fragments, the remaining ions were the same in observed MS/MS spectra of both B.1 and B.2 product. Detection of both products could be attributed to the maximum sensitivity and resolution due to the utilization of nanoUPLC. The hydroxylation of RSV was additionally supported by the neutral loss of water molecule (18.0098 Da) from the precursor ion, *m/z* 500.1865 → *m/z* 482.1767 as is presented in Figure 5. The names of compounds B.1 and B.2 are proposed as 7-(4-(4-fluorophenyl)-6-isopropyl-2-(*N*-methylmethylsulfonamide)pyrimidin-5-yl)-3,5,6-trihydroxyheptanoic acid, and 7-(4-(4-fluorophenyl)-6-isopropyl-2-(*N*-methylmethylsulfo namide)pyrimidin-5-yl)-3,5,7-trihydroxyheptanoic acid, respectively.

#### 2.3.3. Degradation Products C.1/C.2 (*m/z* 498.1710)

**Figure 6:**
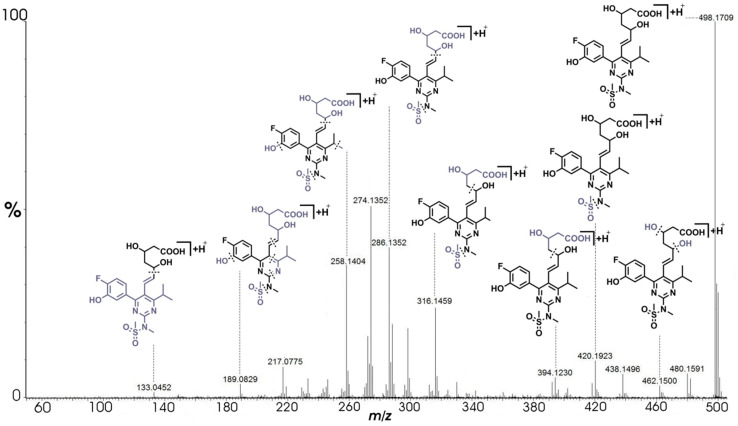
Fragmentation MS/MS spectra of product C.1 [M + H]^+^ ion (precursor ion mass *m/z* 498.1709) obtained by using collision energy of 25 V.

**Scheme 3:**
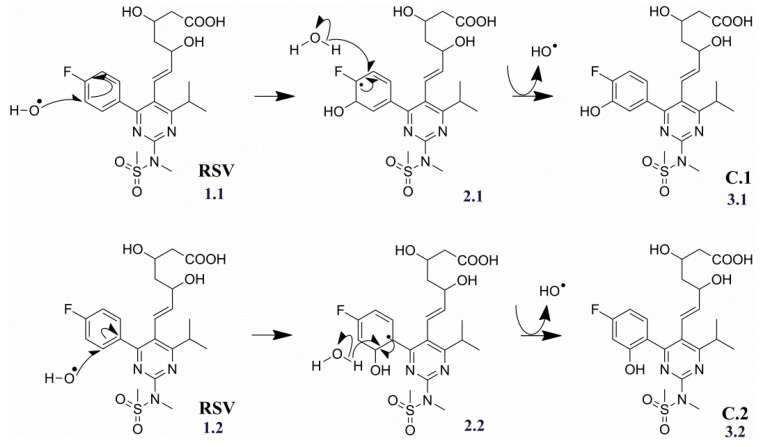
Proposed mechanism of the compounds C.1/C.2 formation by radical substitution on RSV.

Compounds C.1 and C.2 were eluted and detected at RRt 0.88 and RRt 0.94, respectively, as it is shown in TIC (Figure 2). Accompanied MS spectra showed the presence of [M + H]^+^ ion signals at *m/z* 498.1709. The products C.1/C.2 were previously described in the literature, but mechanisms of their formation were not proposed and the position of the hydroxyl substituents in the fluorophenyl ring was not determined [29]. Elemental composition analyses of isomers C.1/C.2 were matched to the molecular formula C_22_H_29_N_3_O_7_FS. The identical MS/MS spectra of both products showed an average mass increment of 15.9943 Da in comparison to the fragmentation pattern of RSV (Figure 6). The mass increment can be explained by the addition of hydroxyl group to the fluorophenyl ring of RSV molecule. The fragment ions observed at *m/z* 133.0452, 189.0829, and 258.1404 were identical to the ions observed in mass spectra obtained from non-irradiated RSV sample. The named three fragments did not comprise structural information about addition of hydroxyl group to the fluorophenyl of RSV molecule. The mechanism assumes that C.1 and C.2 were formed by substitution of the hydrogen atom present in the fluorophenyl ring in the *ortho-* (Scheme 3 (2.1)) and *meta-* (Scheme 3 (2.2)) position by the hydroxyl radical. Spectra obtained after MS/MS analysis of products C.1 and C.2 did not indicate diagnostic ions and therefore did not distinguish the position of attached hydroxyl group to the fluorophenyl group of the RSV molecule. However, fragmentation pathway and ion transition *m/z* 286.1352 → *m/z* 258.1404 clearly pointed out hydroxyl group incorporation in the the fluorophenyl ring. In the literature, similar reactivity of hydroxyl radical towards chlorobenzene was previously reported [40]. Also, the ratio of the regioisomers formed in the reaction with chlorobenzene was similar to the ratio of C.1/C.2 which was found to be equal to 3/1 (Figure 2). The formation of C.1 as a proposed major isomer indicates that the inductive effect of fluorine is more efficient in the stabilization of intermediate radical formed in the substitution reaction than the resonance effect provided by the RSV pyrimidine ring. Resulted products C.1 and C.2 can be named as (*E*)-7-(4-(4-fluoro-3-hydroxyphenyl)-6-isopropyl- 2-(*N*-methylmethylsulfonamide) pyrimidin-5-yl)-3,5-dihydroxyhept-6-enoic acid for C.1 and (*E*)-7-(4-(4-fluoro-2- hydroxyphenyl)-6-isopropyl-2-(*N*-methylmethylsulfonamide) pyrimidin-5-yl)-3,5-dihydroxyhept-6-enoic acid for compound C.2.

#### 2.3.4. Degradation Products D.1/D.2 (*m/z* 514.1659)

**Figure 7:**
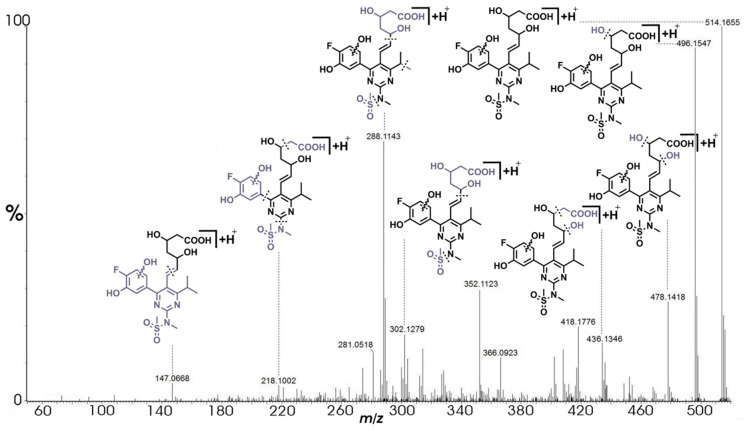
Fragmentation MS/MS spectra of product D.1 [M + H]^+^ ion (precursor ion *m/z* 514.1655) obtained by using collision energy of 25 V.

Compounds D.1 and D.2 were eluted and detected at RRt 0.78 and RRt 0.84, respectively, as it is shown in TIC (Figure 2). In the MS spectra, signals derived from [M + H]^+^ ions were observed at *m/z* 514.1655. This corresponds to elemental composition C_22_H_29_N_3_O_8_FS, the same for both products. Precursor ions of D.1/D.2 relative to products C.1/C.2 and RSV were characterized by a mass increment of 15.9946 Da and 31.9895 Da, respectively. Besides, fragment ions signals observed at *m/z* 496.1547, 478.1418, 436.1346, and 302.1279 showed the same mass shift of 15.9949, on average, compared to the MS/MS fragment ions signals of C.1/C.2 (Figure 7). The shifted fragment ions signals showed the same mass increment relative to the estimated relationship between C.1/C.2 and RSV, which indicates the presence of two hydroxyl groups in the fluorophenyl moieties in the structures of D.1/D.2. Therefore D.1/D.2 can be considered as derivatives of C.1/C.2, resulting from the substitution of a hydrogen atom in the fluorohydroxyphenyl ring with a second hydroxyl radical. As can be observed in Figure 2, one of the double-substituted products was formed in significantly higher quantity relative to the other. After D.1/D.2 MS/MS and spectral differential display analysis, it was not possible to establish exact position of the second hydroxyl group in the fluorohydroxyphenyl ring or to pinpoint diagnostic peaks for specific compound. It can only be supposed, based on the ratio of C.1/C.2 formation, that position next to the fluoro substituent is more favorable for a hydroxyl radical attack than other positions in the benzene ring. The mechanism responsible for the formation of D.1/D.2 from C.1/C.2 is most likely analogous to that proposed for giving rise C.1/C.2 from RSV. Chemical name of product D.1 was proposed as (*E*)-7-(4-(4-fluoro-3,X-dihydroxyphenyl)-6-isopropyl-2-(*N*-methylmethylsulfon amide)pyrimidin-5-yl)-3,5-dihydroxyhept-6-enoic acid, and of product D.2 as (*E*)-7-(4-(4-fluoro-2,X-dihydroxyphenyl)-6-isopropyl-2-(*N*-methylmethylsulfonamide)pyrimidin-5-yl)-3,5-dihydroxyhept-6-enoic acid.

#### 2.3.5. Degradation Product E (*m/z* 480.1605)

**Figure 8:**
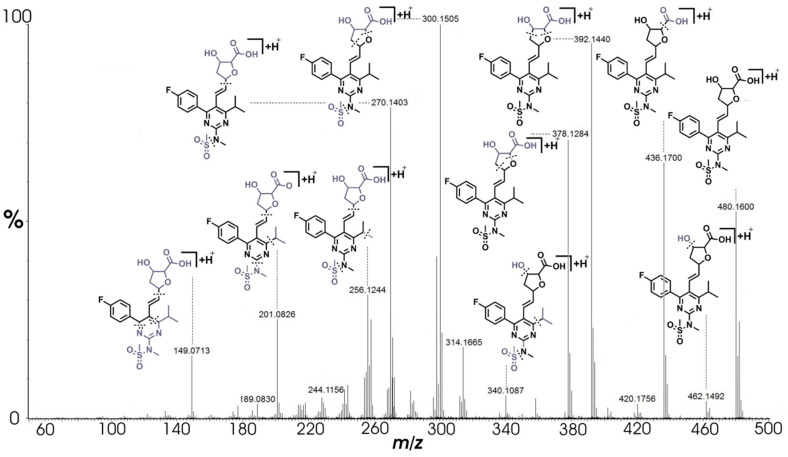
Fragmentation MS/MS spectra of product E [M + H]^+^ ion (precursor ion mass *m/z* 480.1600) obtained by using collision energy of 25 V.

**Scheme 4:**
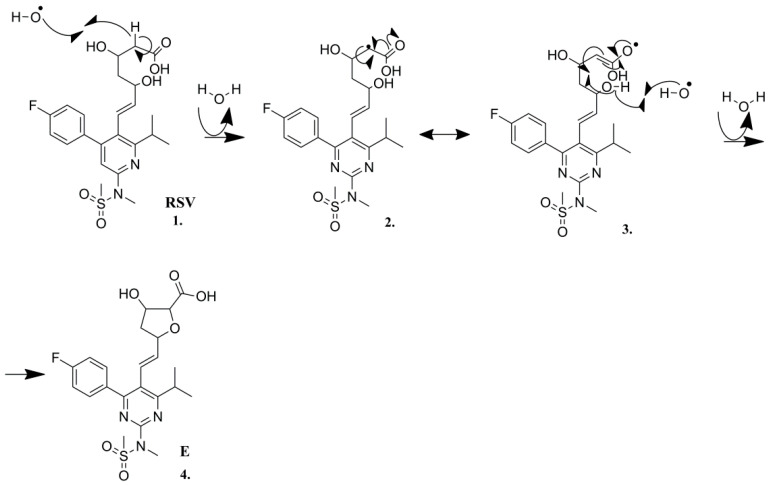
Mechanism of the product E formation by radical-induced cyclization of RSV.

It was determined from TIC that product E eluted at RRt 1.09 (Figure 2). The compound [M + H]^+^ ion signal was observed at *m/z* 480.1600, which corresponded to molecular formula C_22_H_27_N_3_O_6_FS. Fragment ions signals observed in the product MS/MS spectra at *m/z* 189.0830, 300.1505, and 378.1284 (Figure 8) were also seen in the fragmentation pathway of RSV (Figure 3). The formation of a compound with the same molecular formula as found for E was previously reported, but the structure of the compound and the mechanism for its formation were not proposed [29]. Nevertheless, it was found that the product structure had a higher ring and double bond equivalent (RDB) value compared to this one of RSV (10.5 vs 9.5). Herein we propose that the product E is formed in the radical intramolecular cyclization (Scheme 4). The process is initiated by an attack of a hydroxyl radical which abstracts hydrogen atom attached to α carbon atom of the RSV heptanoic acid side chain (Scheme 4 (1)). This leads to the formation of a resonance stabilized intermediate (Scheme 4 (2)). In the next step, the abstraction of the hydrogen atom of the chain δ hydroxyl group (Scheme 4 (3)) initiates an intramolecular cyclization leading to the formation of the substituted tetrahydrofuran ring (Scheme 4 (4)). The presence of the additional ring can explain the higher RDB value found for E in comparison to RSV. Support for the structure proposed for E was also provided by the fragment ions signals observed in the compound MS/MS spectra at *m/z* 462.1492 (loss of hydroxyl group), 436.1700 (loss of carboxylic group), 392.1440, 378.1284, 300.1505, 270.1403 (tetrahydrofuran ring cleavages) and *m/z* 256.1244, 201.0826, and 149.0713 (dissociation of the entire substituted tetrahydrofuran ring). Corresponding molecular formula and structure of product E was proposed as (*E*)-5-(2-(4-(4- fluorophenyl)-6-isopropyl-2-(*N*-methylmethylsulfonamide)pyrimidin-5-yl)vinyl)-3-hydroxytetrahydrofuran-2-carboxylic acid.

#### 2.3.6. Degradation Product F (*m/z* 436.1706)

**Figure 9:**
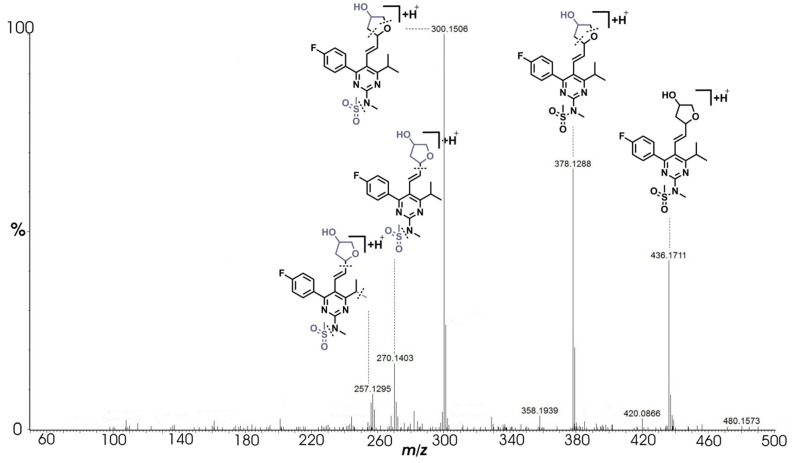
Fragmentation MS/MS spectra of product F [M + H]^+^ ion (product ion mass *m/z* 436.1711) obtained by using collision energy of 45 V.

**Scheme 5:**
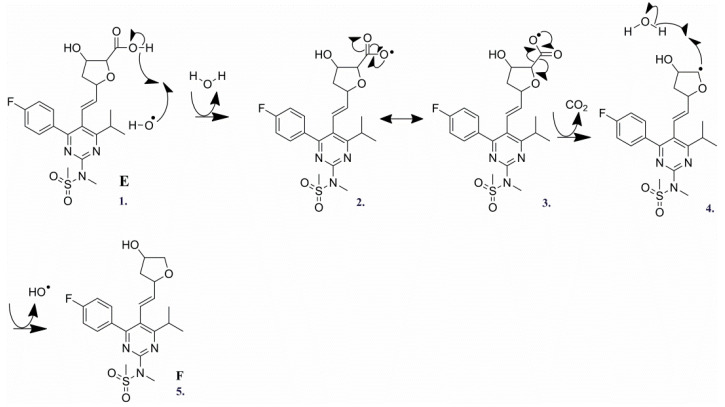
Mechanism of the product F formation by decarboxylation of product E.

Compound F was eluted and detected at RRt 1.21 in TIC (Figure 2). The compound [M + H]^+^ ion signal observed at *m/z* 436.1711 was matched to the molecular formula C_21_H_27_N_3_O_4_FS. Based on results derived from the compound MS/MS spectra (Figure 9) and elemental composition analysis, it was concluded that product F was most likely formed by decarboxylation of compound E (Scheme 5). It is worth noticing that collision energy needed for the induction of the compound fragmentation was equal to 45 V, which is approximately two times higher than collision energy used for product E fragmentation. The cyclization of statins was previously described in a paper dealing with the influence of gamma irradiation on fluvastatin. In that work, it was suggested that the tetrahydrofuran derivative of fluvastatin is formed by the loss of CO_2_ followed by cyclization [23]. Contrary to this, we propose the reverse order of the processes. We suggest that first, as a result of cyclization, product E is formed, the decarboxylation of which leads to the formation of compound F. The name of product F was proposed as (*E*)-*N*-(4-(4-fluorophenyl)-5-(2-(4-hydroxytetrahydrofuran-2-yl)vinyl)-6-isopropylpyrimidin-2-yl)-*N*-methylmethanesulfonamide.

## 3. Materials and Methods

### 3.1. Raw Materials

Analytical grade rosuvastatin calcium (>99%), sodium dihydrogen phosphate (≥99.9%), formic acid (>98%), leucine enkephalin (>98%), and isopropanol (>99%) were obtained from Sigma-Aldrich (St. Louis, MO, USA). High purity nitrous oxide was purchased from Messer Croatia Plin (Zaprešić, Croatia) and acetonitrile (≥99.9%) from Merck Millipore (Burlington, MA, USA). Ultrapure water (18 MΩ·cm) was generated in-house using a Milli-Q System from Merck Millipore (Burlington, MA, USA).

### 3.2. Sample Preparation and Irradiation

A stock solution of RSV in the concentration of 0.1 mg/mL was prepared in the sodium dihydrogen phosphate buffer solution (pH 6.5; 0.01 M). A volume of 3 mL of stock solution was then saturated with nitrous oxide for 15 min at a flow rate of 1 mL/min. The solution prepared was irradiated using panoramic ^60^Co source situated in the Radiation Chemistry and Dosimetry Laboratory at the Ruđer Bošković Institute. RSV sample solutions were subjected to gamma radiation at five radiation doses (50, 100, 200, 500, and 1000 Gy) and the dose rate of 5.8 Gy/s. The temperature of the radiation chamber was 18 °C. The radiation dose rate was determined using an ethanol-chlorobenzene (90:10, *v/v*) dosimetry system, according to the standard ISO 51538:2017.

### 3.3. NanoUPLC-NanoESI-QTOF Analysis

Non-irradiated solution of RSV and irradiated samples were separated on a Waters (Milford, MA, USA) nanoAcquity UPLC system equipped with nanoAcquity UPLC 2G-V/M Symetry C18 Trap Column (100 Å, 5 μm, 180 μm × 20 mm) and nanoAcquity UPLC BEH130 C18 Analytical Column (130 Å, 1.7 μm, 100 μm × 100 mm). The injection volume was 3 μL and the column temperature was 40 °C. Mobile phase consisted out of solvent A (0.1% (*v/v*) formic acid) and solvent B (0.005% (*v/v*) formic acid in acetonitrile). Isocratic delivery of solvent A to the trap column was performed at a flow rate of 15 μL/min for 2 min. Samples were eluted under gradient elution conditions with a flow rate of 1 μL/min and run time of 32 min. The following elution gradient was used: 0–3 min, 80% solvent A; 3–24 min, 45% solvent A; 24–27 min, 1% solvent A; 27–29 min, 80% solvent A; 29–32 min, 80% solvent A. UPLC system was coupled to the nanoESI-QTOF Synapt G2-Si mass spectrometer (Waters, Milford, MA, USA). The instrument parameters were set using the MassLynx software v4.1. (Waters, Milford, MA, USA).

The MS and MS/MS data were collected in high-resolution acquisition mode, covering a mass range between 50 and 1000 Da. Parameters were set as follows: positive ion mode, nitrogen flow of 1.0 bar with a source temperature of 150 °C, the capillary voltage 4.2 kV, and the cone voltage 40 V. The spectral acquisition time was 1 s for MS and 0.5 s for MS/MS analysis. The mass accuracy of the raw data was corrected by infusing 1 ng/μL leucine enkephalin dissolved in isopropanol and 0.1% (*v/v*) formic acid (50:50, *v/v*; 556.2771 Da [M + H]^+^ and 120.0813 Da [M + H]^+^). Leucine enkephalin was infused into the mass spectrometer at the flow rate of 0.4 µL/min every 1 min to ensure high mass accuracy. The collision energy of 25 V was applied for all precursor ions, except for product F where collision energy of 45 V was applied.

Elemental composition analysis was performed in duplicates to fit and calculate unambiguous molecular formulas. The raw data were processed with MassLynx v4.1 (software equipped with isotope fitting algorithm MassFragment). Accurate mass measurement (<2 ppm), isotope pattern-based implementation (i-FIT algorithm), ring and double bond equivalent (RDB) 1.5–50, and 0–500 C, 0–1000 H, 0–6 S, 0–2 F, 0–200 O, 0–200 N, 0–8 Cl and 0–8 Br were used in elemental composition analysis for identification and confirmation of precursor and product ion fragments.

## 4. Conclusions

In this study, degradation products of gamma-induced irradiation of RSV are described for the first time. Overall nine degradation products are described of which four were correlated to the same or similar products generated by ZnO photocatalysis of RSV [29]. Remaining five products have never been described in the literature before this study. The essential part of unknown structure identification of degradation products was performed using a rapid and sensitive method based on nanoUPLC and nanoESI-QTOF. The named method enabled accurate mass analysis of products and precursor ions (<2 ppm) which was necessary for unambiguous structural identification and understanding of associated radical formation mechanisms. It was deduced that in addition to cyclization and decarboxylation caused by ^●^OH radical interactions (products E and F), ^●^OH radicals are attracted primarily to the conjugated part of a molecule with an excess of electrons (products A, B.1/B.2, C.1/C.2, and D.1/D.2). The revealed interactions between rosuvastatin and degradation product molecules, and ^●^OH radicals provide valuable information for future research concerning synthesis of new statin molecules, as well as understanding the mechanism of antioxidant action of RSV which has so far been only a hypothesis. Furthermore, since gamma radiation is considered as a possible method for wastewater remediation, our study confirms the degradation of rosuvastatin by applying small doses of radiation.

## Figures and Tables

**Table 1:** nanoLC-nanoESI-QTOF/MS data of RSV and degradation product [M + H]^+^ ions and corresponding MS/MS data. The asterisk is used to denote diagnostic ions present only in specific isobaric MS/MS spectra.

Products	Molecular Formulae	Experimental Mass (Da)	Theoretical Mass (Da)	Mass Error (ppm)	RDB	Major Fragments (Chemical Formula)
RSV	C_22_H_29_N_3_O_6_FS+	482.1760	482.1761	−0.21	9.5	464.1653 (C_22_H_27_N_3_O_5_FS+), 446.1553 (C_22_H_25_N_3_O_4_FS+), 422.1549 (C_20_H_25_N_3_O_4_FS+), 404.1984 (C_21_H_27_N_3_O_4_F+), 378.1282 (C_18_H_21_N_3_O_3_FS+), 376.1484 (C_19_H_23_N_3_O_2_FS+), 314.1665 (C_18_H_21_N_3_OF+) 300.1508 (C_17_H_19_N_3_OF+), 282.1403 (C_17_H_17_N_3_F+), 272.1559 (C_16_H_19_N_3_F+), 270.1409 (C_16_H_17_N_3_F+), 258.1402 (C_15_H_17_N_3_F+), 256.1242 (C_15_H_15_N_3_F+), 230.1093 (C_13_H_13_N_3_F+), 189.0827 (C_11_H_10_N_2_F+), 133.0454 (C_5_H_9_O_4_+)
A	C_22_H_30_N_3_O_7_S+	480.1806	480.1805	0.21	9.5	462.1712 (C_22_H_28_N_3_O_6_S+), 444.1599 (C_22_H_26_N_3_O_5_S+), 420.1600 (C_20_H_26_N_3_O_5_S+), 402.2024 (C_21_H_28_N_3_O_5_+), 376.1327 (C_18_H_22_N_3_O_4_S+), 312.1708 (C_18_H_22_N_3_O_2_+), 298.1554 (C_17_H_20_N_3_O_2_+), 280.1446 (C_17_H_18_N_3_O+), 270.1600 (C_16_H_20_N_3_O+), 268.1446 (C_16_H_18_N_3_O+), 256.1446 (C_15_H_18_N_3_O+), 254.1293 (C_15_H_16_N_3_O+), 199.0873 (C_12_H_11_N_2_O+), 187.0870 (C_11_H_11_N_2_O+), 131.0496 (C_9_H_7_O+)
B.1/B.2	C_22_H_31_N_3_O_7_FS+	500.1865	500.1867	−0.40	8.5	482.1767 (C_22_H_29_N_3_O_6_FS+), 464.1646 (C_22_H_27_N_3_O_5_FS+), 446.1538 (C_22_H_25_N_3_O_4_FS+), 422.1540 (C_20_H_25_N_3_O_4_FS+), 404.1974 (C_21_H_27_N_3_O_4_F+), 378.1268 (C_18_H_21_N_3_O_3_FS+), 376.1470 (C_19_H_23_N_3_O_2_FS+), 350.1330 (C_17_H_21_N_3_O_2_FS+), 300.1502 (C_17_H_19_N_3_OF+), 288.1495 (C_16_H_19_N_3_OF+), 281.0492 (C_11_H_11_N_3_O_4_S+), 270.1400 (C_16_H_17_N_3_F+), 258.1398 (C_15_H_17_N_3_F+), 256.1243 (C_15_H_15_N_3_F+), 228.0807 (C_9_H_14_N_3_O_2_S+), 201.0836 (C_12_H_10_N_2_F+), 189.0823 (C_11_H_10_N_2_F+), 175.0736 * (C_9_H_9_N_3_O+), 147.0663 * (C_6_H_11_O_4_+), 133.0459 (C_5_H_9_O_4_+)
C.1/C.2	C_22_H_29_N_3_O_7_FS+	498.1709	498.1710	−0.20	9.5	480.1595 (C_22_H_27_N_3_O_6_FS+), 462.1502 (C_22_H_25_N_3_O_5_FS+), 438.1485 (C_20_H_25_N_3_O_5_FS+), 420.1923 (C_21_H_27_N_3_O_5_F+), 394.1230 (C_18_H_21_N_3_O_4_FS+), 342.1599 (C_19_H_21_N_3_O_2_F+), 317.1476 (C_17_H_20_N_3_O_2_F+), 316.1459 (C_17_H_19_N_3_O_2_F+), 298.1350 (C_17_H_17_N_3_OF+), 296.1195 (C_17_H_15_N_3_OF+), 288.1506 (C_16_H_19_N_3_OF+), 287.1384 (C_16_H_18_N_3_OF+), 286.1352 (C_16_H_17_N_3_OF+), 274.1350 (C_15_H_17_N_3_OF+), 273.1260 (C_15_H_16_N_3_OF+), 272.1192 (C_15_H_15_N_3_OF+), 258.1397 (C_15_H_17_N_3_F+), 246.1033 (C_13_H_13_N_3_OF+), 217.0767 (C_12_H_10_N_2_OF+), 189.0838 (C_10_H_11_N_3_O+)
D.1/D.2	C_22_H_29_N_3_O_8_FS+	514.1655	514.1659	−0.78	9.5	496.1547 (C_22_H_27_N_3_O_7_FS+), 478.1418 (C_22_H_25_N_3_O_6_FS+), 452.1666 (C_21_H_27_N_3_O_5_FS+), 436.1880 (C_21_H_27_N_3_O_6_F+), 436.1346 (C_20_H_23_N_3_O_5_FS+), 418.1776 (C_21_H_25_N_3_O_5_F+), 408.1402 (C_19_H_23_N_3_O_4_FS+), 402.1820 (C_21_H_25_N_3_O_4_F+), 400.1643 (C_21_H_23_N_3_O_4_F+), 366.0923 (C_16_H_17_N_3_O_4_FS+), 352.1123 (C_16_H_19_N_3_O_3_FS+), 328.1451 (C_18_H_19_N_3_O_2_F+), 314.1311 (C_17_H_17_N_3_O_2_F+), 302.1279 (C_16_H_17_N_3_O_2_F+), 289.1178 (C_15_H_16_N_3_O_2_F+), 288.1143 (C_15_H_15_N_3_O_2_F+), 281.0518 (C_11_H_11_N_3_O_4_S+), 274.1363 (C_15_H_17_N_3_OF+), 265.0165 (C_10_H_7_N_3_O_4_S+), 218.1002 (C_12_H_14_N_2_O_2_+), 147.0668 (C_6_H_11_O_4_+)
E	C_22_H_27_N_3_O_6_FS+	480.1600	480.1605	−1.04	10.5	462.1492 (C_22_H_25_N_3_O_5_FS+), 436.1700 (C_21_H_27_N_3_O_4_FS+), 420.1756 (C_21_H_27_N_3_O_3_FS+), 392.1440 (C_19_H_23_N_3_O_3_FS+), 378.1284 (C_18_H_21_N_3_O_3_FS+), 358.1205 (C_18_H_17_N_3_O_4_F+), 340.1087 (C_18_H_15_N_3_O_3_F+), 314.1665 (C_18_H_21_N_3_OF +), 300.1505 (C_17_H_19_N_3_OF+), 298.1350 (C_17_H_17_N_3_OF+), 282.1402 (C_17_H_17_N_3_F+), 270.1403 (C_16_H_17_N_3_F+), 270.1042 (C_15_H_13_N_3_OF+), 256.1244 (C_15_H_15_N_3_F+), 201.0826 (C_12_H_10_N_2_F+), 189.0830 (C_11_H_10_N_2_F+), 189.0830 (C_12_H_13_O_2_+), 177.1034 (C_10_H_13_N_2_O+), 149.0713 (C_10_H_10_F+)
F	C_21_H_27_N_3_O_4_FS+	436.1711	436.1707	0.92	9.5	378.1288 (C_18_H_21_N_3_O_3_FS+), 300.1506 (C_17_H_19_N_3_OF+), 270.1403 (C_16_H_17_N_3_F+), 257.1295 (C_15_H_16_N_3_F+)

## Data Availability

The data presented in this study are openly available in Mendeley Data at doi: 10.17632/cg3hmww4t3.1, reference number [41].

## Supplementary Materials

The following are available online at https://www.mdpi.com/article/10.3390/ph14111160/s1, Figure S1: Total Ion Current (TIC) chromatograms of irradiated (A) and non-irradiated RSV samples (B).
