# Supplementary figures and images for: NanoUPLC-QTOF-MS/MS Determination of Major Rosuvastatin Degradation Products Generated by Gamma Radiation in Aqueous Solution
Source: Pharmaceuticals (Basel). 2021 Nov 13;14(11):1160. doi: 10.3390/ph14111160 (PMC8622667; doi:10.3390/ph14111160)

**Figure S1.** Total Ion Current (TIC) chromatograms of irradiated (A) and non-irradiated RSV samples (B).

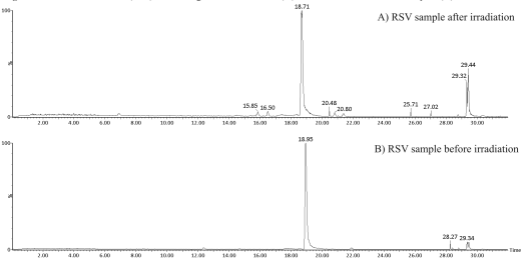

Supplement: Supplementary file 1 [file pharmaceuticals-14-01160-s001.zip › pharmaceuticals-1377334-supplementary.pdf]
